# Natural Amyloid-Beta Oligomers Acutely Impair the Formation of a Contextual Fear Memory in Mice

**DOI:** 10.1371/journal.pone.0029940

**Published:** 2012-01-04

**Authors:** Kara A. Kittelberger, Fabrizio Piazza, Giuseppina Tesco, Leon G. Reijmers

**Affiliations:** 1 Department of Neuroscience, Tufts University School of Medicine, Boston, Massachusetts, United States of America; 2 Alzheimer's Disease Research Laboratory, Department of Neuroscience, Tufts University School of Medicine, Boston, Massachusetts, United States of America; Federal University of Rio de Janeiro, Brazil

## Abstract

Memory loss is one of the hallmark symptoms of Alzheimer's disease (AD). It has been proposed that soluble amyloid-beta (Abeta) oligomers acutely impair neuronal function and thereby memory. We here report that natural Abeta oligomers acutely impair contextual fear memory in mice. A natural Abeta oligomer solution containing Abeta monomers, dimers, trimers, and tetramers was derived from the conditioned medium of 7PA2 cells, a cell line that expresses human amyloid precursor protein containing the Val717Phe familial AD mutation. As a control we used 7PA2 conditioned medium from which Abeta oligomers were removed through immunodepletion. Separate groups of mice were injected with Abeta and control solutions through a cannula into the lateral brain ventricle, and subjected to fear conditioning using two tone-shock pairings. One day after fear conditioning, mice were tested for contextual fear memory and tone fear memory in separate retrieval trials. Three experiments were performed. For experiment 1, mice were injected three times: 1 hour before and 3 hours after fear conditioning, and 1 hour before context retrieval. For experiments 2 and 3, mice were injected a single time at 1 hour and 2 hours before fear conditioning respectively. In all three experiments there was no effect on tone fear memory. Injection of Abeta 1 hour before fear conditioning, but not 2 hours before fear conditioning, impaired the formation of a contextual fear memory. In future studies, the acute effect of natural Abeta oligomers on contextual fear memory can be used to identify potential mechanisms and treatments of AD associated memory loss.

## Introduction

Alzheimer's disease (AD) is characterized by a severe loss of memory function. It has been proposed that AD associated memory loss is caused by soluble amyloid-beta (Abeta) oligomers, especially during early stages of AD before significant neuronal cell death has occurred [1], [2]. This model is supported by a number of observations. First, soluble Abeta levels, but not plaque number or insoluble Abeta levels, correlate with the severity of AD [3], [4]. Second, in AD mouse models memory loss is observed before the formation of plaques but during and correlating with an increase in Abeta oligomers [5], [6], [7]. Third, Abeta oligomers isolated from AD brains can impair memory when injected in rodent brains [8]. Interestingly, the effects of Abeta oligomers on memory are acute and reversible [7], [8], [9]. This suggests that, at least during the early stages of AD, memory loss might be reversed by preventing the formation or action of Abeta oligomers.

It is not clear how Abeta oligomers impair memory. Extracellular Abeta oligomers are able to bind to neurons, and a number of molecules have been proposed as binding sites for Abeta oligomers [10], [11], [12]. There is an ongoing effort to determine which of these binding sites are essential for the cognitive effects of Abeta oligomers [13], [14]. Despite uncertainty about the critical binding site(s), there is growing consensus that binding of Abeta oligomers to neurons in the hippocampus impairs synaptic plasticity [8], [10], [15], [16]. Impairment of hippocampal synaptic plasticity by Abeta oligomers is an attractive candidate mechanism for AD associated memory loss, which is characterized by an early and severe loss of hippocampus dependent memories.

The aim of this study was to test if natural Abeta oligomers can impair fear conditioning in mice. We use the term natural Abeta oligomers to refer to Abeta oligomers that are produced by cells, as opposed to synthetic Abeta oligomers that are produced in-vitro. The source of Abeta is an important consideration [1], [17]. For example, natural Abeta oligomers can impair memory and long-term potentiation at doses a hundred to thousand times lower than effective doses of synthetic Abeta oligomers [18], [19]. Fear conditioning is impaired in AD patients [20], [21], and in transgenic AD mouse models [22]. In order to further test if soluble Abeta oligomers play a causative role in AD associated memory loss, it is important to verify that impaired fear conditioning can be caused by natural Abeta oligomers. We therefore tested the effects of natural Abeta oligomers on two types of memory that result from fear conditioning, one dependent on the hippocampus (contextual fear memory) and one independent from the hippocampus (tone fear memory) [23]. We found that natural Abeta oligomers acutely impaired the formation of a contextual fear memory.

## Results

### Natural Abeta oligomer solution and injection

**Figure 1:**
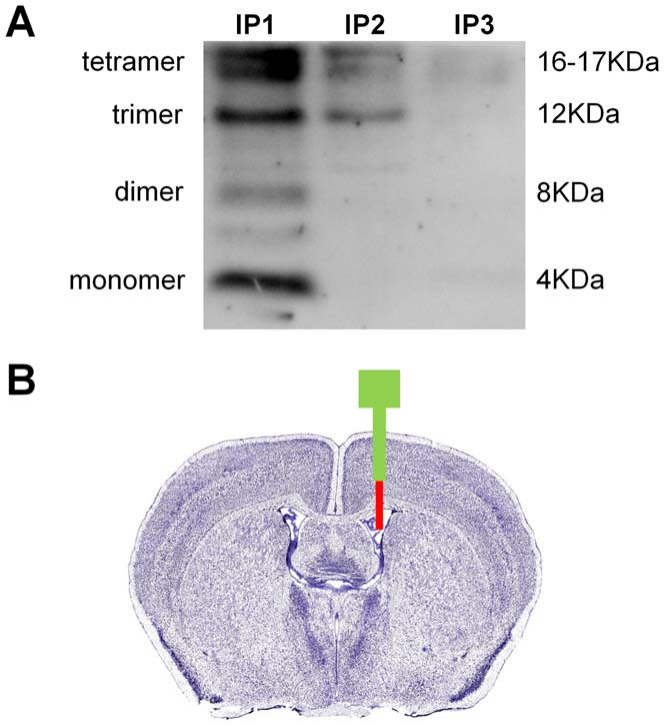
Natural Abeta oligomer solution and injection. A) Blot image showing the presence of Abeta monomers, dimers, trimers, and tetramers in the Abeta solution. The 6E10 antibody was used for detection of Abeta oligomers that were removed from the Abeta solution using immunoprecipitation with A/G beads and 4G8 antibody (IP1, IP2, IP3: oligomers bound to beads used for first, second, and third immunoprecipitation). No oligomers were detected after three rounds of immunoprecipitation, which confirmed the absence of Abeta oligomers in the control solution (see “Materials and Methods” for a detailed description of how Abeta and control solutions were generated). B) Diagram showing the location of the guide cannula (green) and the injector cannula (red) in a Nissl-stained coronal section of the mouse brain [38]. The tip of the guide cannula stopped just above the corpus callosum. The tip of the injection cannula extended into the lateral ventricle.

Figure 1A shows that the natural Abeta oligomer solution used in this study contained a mixture of Abeta monomers, dimers, trimers, and tetramers. After three rounds of immunodepletion no Abeta oligomers could be detected, confirming the absence of Abeta oligomers in the control solution. The Abeta oligomer solution also contained secreted amyloid-precursor protein (sAPP). However, sAPP was still present after immunodepletion (figure S1). Therefore, any differences between the effects of the Abeta oligomer solution and the control solution had to be caused by Abeta oligomers. The Abeta oligomer solution contained between 12,000–14,000 pg/ml of total Abeta42 as measured by ELISA. This concentration is comparable to similar preparations used in earlier studies, and comparable to Abeta detected in human cerebrospinal fluid [15]. Figure 1B shows the location in the lateral brain ventricle where Abeta and control solutions were injected.

### Experiment 1: repeated Abeta injection

**Figure 2:**
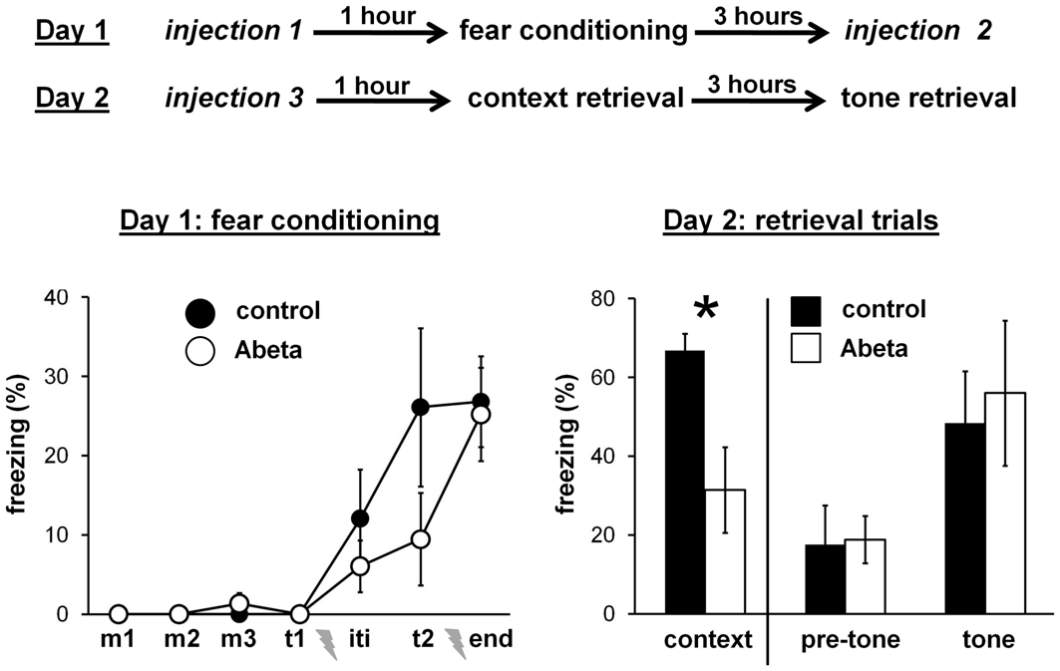
Experiment 1: repeated Abeta injection. Top) Diagram showing the design of experiment 1. Separate groups of mice were injected three times with either control or Abeta solution. Bottom) Graphs showing average freezing scores during fear conditioning on day 1 and the two retrieval trials on day 2 (see “Materials and Methods: Analysis of freezing behavior” for explanation of intervals on the X axis). Mice injected with the Abeta solution (n = 5) had significantly lower freezing scores during the context fear retrieval trial as compared with mice injected with the control solution (n = 6). Error bars are standard errors of means. * *P*<0.05.

Figure 2 shows the design and results of experiment 1. Repeated injection of Abeta oligomers had no effect on baseline freezing or fear conditioning, as indicated by similar freezing levels for both groups at the start and end of fear conditioning on day 1. Repeated Abeta injection resulted in a significantly lower freezing score on day 2 during the context retrieval trial (*t*(9) = 3.25, *P* = 0.010), indicating an impairment in contextual fear memory. There was no difference between the 2 groups during the tone retrieval trial, with similar freezing scores before and after the onset of the tone. The data show that natural Abeta oligomers induced a specific impairment in contextual fear memory, and that they did not affect tone fear memory.

### Experiment 2: single Abeta injection 1 hour before fear conditioning

**Figure 3:**
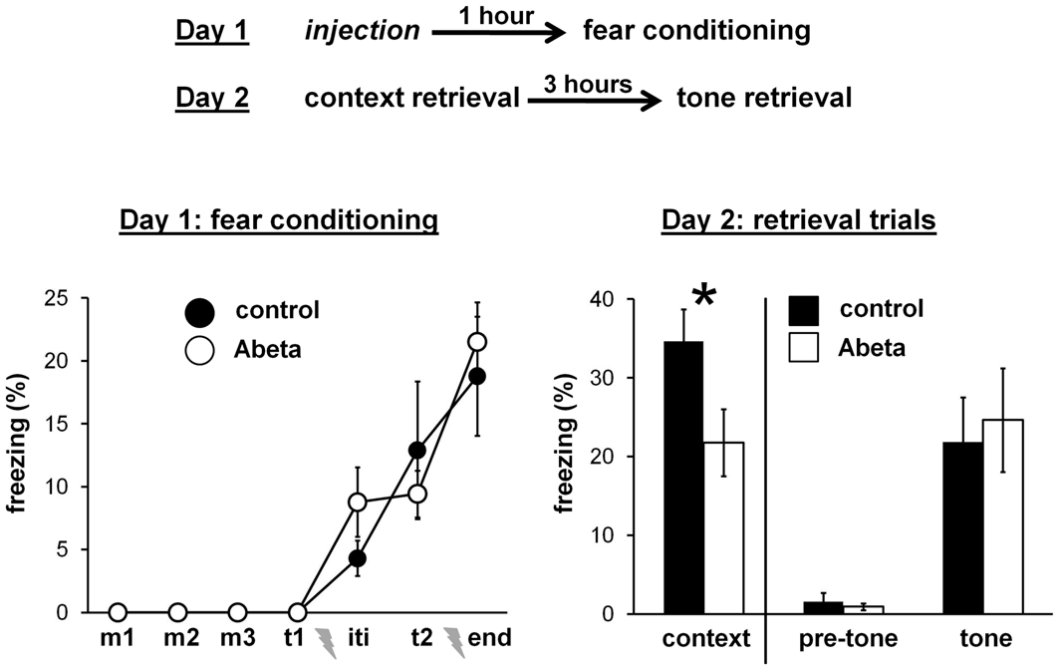
Experiment 2: single Abeta injection 1 hour before fear conditioning. Top) Diagram showing the design of experiment 2. Separate groups of mice were injected one time with either control or Abeta solution 1 hour before fear conditioning. Bottom) Graphs showing average freezing scores during fear conditioning on day 1 and the two retrieval trials on day 2. Mice injected with the Abeta solution (n = 8) had significantly lower freezing scores during the context fear retrieval trial as compared with mice injected with the control solution (n = 10). Error bars are standard errors of means. * *P*<0.05.

Figure 3 shows the design and results of experiment 2. Injection of Abeta 1 hour before fear conditioning had no effect on baseline freezing or fear conditioning on day 1. On day 2, the Abeta injected group had a significantly lower average freezing score during the context retrieval trial compared with the control group (*t*(16) = 2.18, *P* = 0.044). No effect was found on freezing during the tone retrieval trial. Again, similar to experiment 1, natural Abeta oligomers impaired contextual fear memory but not tone fear memory.

### Experiment 3: single Abeta injection 2 hours before fear conditioning

**Figure 4:**
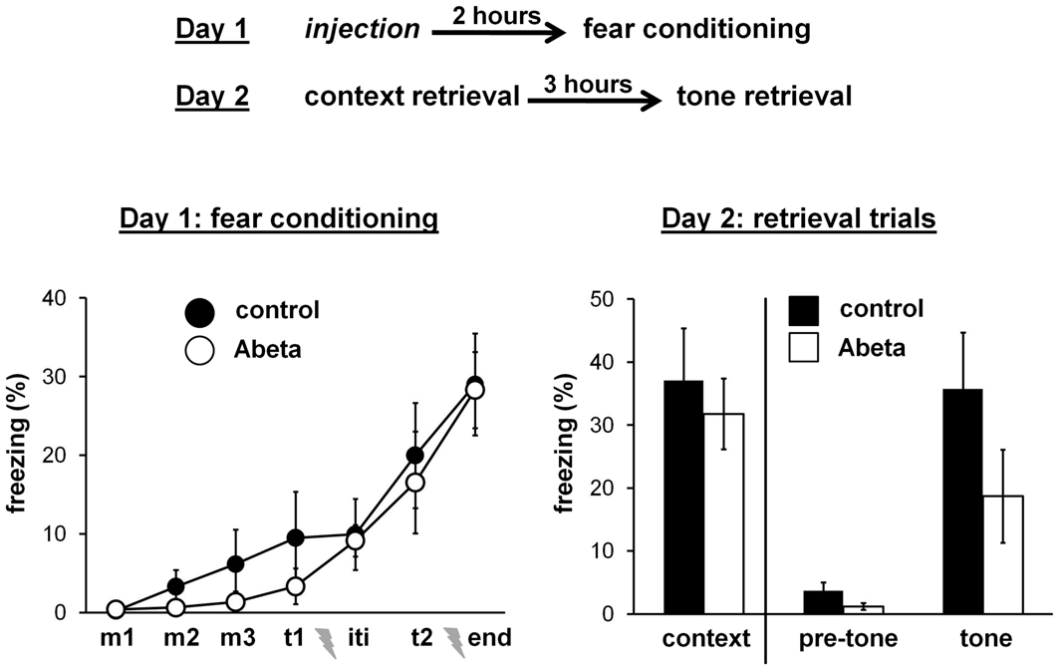
Experiment 3: single Abeta injection 2 hours before fear conditioning. Top) Diagram showing the design of experiment 3. Separate groups of mice were injected one time with either control or Abeta solution 2 hours before fear conditioning. Bottom) Graphs showing average freezing scores during fear conditioning on day 1 and the two retrieval trials on day 2. There was no difference during any of the intervals analyzed between mice injected with the Abeta solution (n = 10) and mice injected with the control solution (n = 8). Error bars are standard errors of means.

Figure 4 shows the design and results of experiment 3. Injection of Abeta 2 hours before fear conditioning had no effect on fear conditioning on day 1. There was no effect on the retrieval of the context fear memory and the retrieval of the tone fear memory on day 2. The data show that natural Abeta oligomers injected 2 hours before fear conditioning did not impair contextual and tone fear memories.

### Effects of control injections

Figure S2 shows a comparison between the three control groups from experiment 1–3 and a group of mice that was not subjected to surgery and injection. Repeated control injections increased freezing during the context retrieval trial (figure S2A; *t*(10) = 3.79, *P* = 0.004). Therefore, for future experiments single injection might be preferred, or alternatively, habituation to injection might be required to prevent possible stress effects caused by repeated injections. Importantly, the surgery and control solution had no effect on either contextual fear memory or tone fear memory, as indicated by similar freezing scores for mice injected once with a single control injection and mice that were not subjected to surgery and injection (figure S2B–C).

## Discussion

The main conclusion from this study is that natural Abeta oligomers can acutely impair the formation of a contextual fear memory. This conclusion is supported by three experiments that used different time points of Abeta injection. The first experiment used repeated injection before and after fear conditioning and before retrieval. This resulted in impaired retrieval of contextual fear memory. In order to determine which time point caused this effect, we performed a second experiment with a single injection 1 hour before fear conditioning. This again resulted in impaired retrieval of contextual fear memory. In order to determine if this was caused by an acute effect on fear conditioning or a delayed effect on context fear retrieval, we performed a third experiment with a single Abeta injection 2 hours before fear conditioning. This had no effect on the retrieval of contextual fear memory, indicating that the single Abeta injection did not have a lasting effect causing impaired retrieval one day later. Therefore, we conclude that injecting Abeta 1 hour before fear conditioning resulted in an Abeta concentration around the time of fear conditioning that acutely impaired the formation of a contextual fear memory. The lack of effect of Abeta injected 2 hours before fear conditioning might have been caused by the reported 2 hour half-life of Abeta in the brain [24]. The impaired retrieval of the contextual fear memory found in experiment 1 and 2 was not caused by a state-dependent effect, meaning the internal state of the mice during retrieval differed from their internal state during fear conditioning. In experiment 1 the mice were injected both before fear conditioning and before retrieval and therefore presumably were in the same state, which nevertheless still resulted in an impaired contextual fear memory. In summary, the data indicate that natural Abeta oligomers have an acute effect during or shortly after fear conditioning, which results in the impaired formation of a contextual fear memory.

An acute effect of Abeta oligomers during or shortly after fear conditioning would impact learning or consolidation respectively. Effects on learning have to be interpreted with caution, since they might be caused by impaired detection of the conditioned stimulus (CS: context) or the unconditioned stimulus (US: footshock). In none of the three experiments we observed an effect of Abeta on the formation and retrieval of a tone fear memory. This shows that Abeta injection did not impair hearing function or shock sensation. In addition, Abeta injection did not impair freezing at the end of the fear conditioning trial when the context was the only available CS. This indicates that Abeta injected mice were able to detect both the CS (context) and the US (footshock). Since Abeta did not impair freezing during fear conditioning, it is likely that learning was unaffected and that the impaired retrieval of the contextual fear memory was caused by impaired consolidation shortly after fear conditioning. However, it can not be excluded that learning was impaired by Abeta in a way that did not affect the immediate expression of fear during fear conditioning. Future studies that inject Abeta right after fear conditioning might be able to distinguish between Abeta effects on learning and consolidation. Either way, our data are in line with previous studies that have reported effects of Abeta oligomers on learning and consolidation [13], [25], [26].

Contextual fear memory is dependent on the hippocampus and tone fear memory is independent from the hippocampus [23], which suggests that the specific impairment of contextual fear memory in our study was caused by Abeta oligomers that diffused from the ventricle into the hippocampus. Accordingly, previous studies found that injection of natural Abeta oligomers into the lateral ventricle impaired synapse remodeling in the hippocampus [27], and increased glutamate levels in the hippocampus [28]. Our inability to detect an effect on tone fear memory might indicate that an insufficient fraction of the injected Abeta reached the amygdala, which is a brain region critical for formation of tone fear memories [23]. Since together with the hippocampus, the amygdala is one of the first brain regions affected in AD patients [29], future studies could inject Abeta oligomers directly into the amygdala to explore a possible role for the amygdala in AD associated memory loss.

Our finding that natural Abeta oligomers acutely impair the formation of a contextual fear memory is in agreement with previous studies that injected synthetic Abeta oligomers into the ventricle or hippocampus [25], [26]. In contrast, another study that injected synthetic Abeta oligomers into the hippocampus reported an improvement of contextual fear memory, suggesting that low levels of synthetic Abeta oligomers might have beneficial effects on memory [30]. Since synthetic and natural Abeta oligomers have different efficacy profiles [18], [19], and no positive effect of natural Abeta oligomers on memory has been reported yet, it remains to be shown if low levels of natural Abeta oligomers can also have positive effects on memory.

The results from our study are in agreement with other data that support a causal role of soluble Abeta oligomers in AD associated memory loss [1], [2]. We used 7PA2 cells to produce a natural Abeta oligomer solution that contained Abeta monomers, dimers, trimers, and tetramers. These same Abeta oligomers were found in 7PA2 preparations used in earlier studies [31], [32], [33] Earlier studies using 7PA2 Abeta oligomers found binding of Abeta oligomers to EphB2 protein, impaired in-vivo long-term potentiation, and impaired memory as tested in the alternating lever cyclic ratio test, radial arm maze, and passive avoidance test [9], [12], [15], [27], [34]. Importantly, the natural Abeta oligomer solution derived from 7PA2 cells used in this and other studies contains Abeta oligomer species similar to those found in AD brain tissue, which when injected into rat brains also impair memory [8]. It is therefore conceivable that injection of 7PA2 derived natural Abeta oligomers into the mouse brain recapitulates a causal factor responsible for memory loss in AD patients, and that the mechanism responsible for Abeta oligomer impaired contextual fear conditioning also contributes to AD associated memory loss.

In summary, we found an acute impairing effect of natural Abeta oligomers on contextual fear memory in mice. This finding is in agreement with natural Abeta oligomer induced memory impairments found in previous studies that used rats [7], [8], [9], [18], [27], [34], [35], [36]. Our data show, to our knowledge, for the first time that natural Abeta oligomers can also impair memory in mice. Our study thereby paves the way for using genetic mouse models to study the underlying mechanisms by which natural Abeta oligomers impair memory. This is also, to our knowledge, the first time that an effect of natural Abeta oligomers on fear conditioning is reported. Since fear conditioning requires only a single learning trial, it is highly suitable for dissecting the different stages of memory (acquisition, consolidation, storage, and retrieval), allowing to test the contribution of each memory stage to AD associated memory loss. We propose that natural Abeta oligomer impaired fear conditioning can be used to test potential mechanisms and treatments of AD associated memory loss. A better understanding of the acute effects of natural Abeta oligomers might lead to treatments that can reverse cognitive impairments during early stages of AD, and possibly prevent or delay the progress of AD.

## Materials and Methods

### Ethics statement

This study was performed in strict accordance with the recommendations in the Guide for the Care and Use of Laboratory Animals of the National Institutes of Health. The protocol was approved by the Institutional Animal Care and Use Committee of Tufts University (Protocol Number: B2009-118). All surgery was performed under xylazine/ketamine anesthesia, and every effort was made to minimize suffering.

### Natural amyloid-beta oligomer and control solutions

A solution containing natural amyloid-beta (Abeta) oligomers was derived from the conditioned medium of 7PA2 cells (a kind gift of Dr. Dennis Selkoe), which are Chinese Hamster Ovary cells stably transfected with cDNA encoding APP751, an amyloid precursor protein that contains the Val717Phe familial Alzheimer's disease mutation [15], [37]. Five million 7PA2 cells were cultured in Dulbecco's modified Eagle's medium (DMEM, Hyclone) containing 10% bovine fetal calf serum (Atlanta Biologicals), 50 µg/ml penicillin/streptomycin, 2 mM L-glutamine (Sigma-Aldrich), and 200 µg/ml G418 (Calbiochem). After 24 h cells were washed with serum-free medium and conditioned in 5 ml of plain DMEM lacking any additives, overnight. The oligomer-containing conditioned medium (CM) was removed and cleared of cells by centrifugation at 200 g for 10 min at 4°C. CM was then concentrated 10-fold by centrifugation at 3500 g for 45 min using Amicon Ultra-15 (Millipore), and this concentrated CM was used as the natural Abeta oligomer solution in the fear conditioning experiments. The concentrated CM contained between 12,000–14,000 pg/ml of Abeta42 as measured by ELISA. A control solution was prepared by immunodepleting the concentrated CM using the Abeta antibody 4G8 (Covance, SIG-39220-200). Concentrated CM (250 microliter) was first pre-cleared with protein A/G beads (Santa Cruz Biotechnology) for 1h at 4°C. Next, three rounds of immunodepletion, 6h each at 4°, were performed using protein A/G beads with 4G8 antibody (45 microliter of beads, 3 microgram of antibody per round). To ensure that all the antibody used for immunodepletion was removed, concentrated CM was post-cleared after the third IP round with protein A/G beads for 1h at 4°C. Complete removal of Abeta oligomers was confirmed by 4–12% BIS-TRIS minigels with MES immunoblotting (Invitrogen) and the Abeta 6E10 detection antibody (Covance, SIG-39320-200). In addition, we confirmed with immunoblotting that the immunodepletion had no effect on the amount of secreted amyloid precursor protein. Aliquots of Abeta and control solutions were stored at −80°C until the day of injection.

### Mice

For all experiments male C57Bl/6J mice were used. Mice were ordered from Jackson Laboratories (Bar Harbor, Maine) at an age of 9 weeks, and allowed to acclimate for at least one week before surgery. Mice were housed in a climate controlled room under a regular light/dark cycle with lights on at 7 am and lights off at 7 pm.

### Surgery

Mice were anesthesized with an intraperitoneal injection of xylazine/ketamine (10 mg/kg xylazine and 100 mg/kg ketamine) and placed in a stereotaxic instrument. An incision was made to expose the skull and one unilateral guide cannula (Plastics One; C315GS-5-SPC, cut 1.5 mm below the pedestal) was implanted just above the dorsal lateral ventricle using stereotaxic coordinates (AP = −0.2 mm, ML = +1.0 mm, DV = −1.5 mm relative to bregma). One anchor screw (CMA Microdialysis; 7431021) and cement (Fisher Scientific; Durelon Triple Cement Kit, NC9332928) was used to attach the guide cannula to the skull. Sutures were used to close the incision, a topical antibiotic was applied, and a dummy was inserted into the guide cannula to prevent clogging (Plastics One; C315DCS-5-SPC, cut to completely fill the guide cannula). After the surgery, mice received subcutaneous injections of buprenorphine (0.1mg/kg) and sterile saline (0.5 ml) for analgesia and rehydration respectively. Mice recovered from the surgery for at least 5 days before injection and behavioral testing started.

### Injection

Aliquots of Abeta and control solutions were thawed, gently tapped, and kept on ice until injection on the same day. Abeta and control solutions were injected into the lateral ventricle while the mice were awake and freely moving. This was achieved by removing the dummy and placing an internal injector cannula (Plastics One; C315IS-5-SPC, cut to extend 1 mm projection below the tip of the guide cannula) into the guide cannula. The injector cannula was connected by tubing (Plastics One; C313C, 40 cm) with a syringe (Hamilton; 701RN, 10 microliter with removable blunt 22 gauge needle). Tubing was filled with sterile water to enable sufficient pressure for injection. Abeta and control solutions were drawn into the injector cannula and separated from the sterile water in the tubing with an air bubble. All injections had a 2 microliter volume and were administered over a 5 minute period (400 nanoliter/minute) using an electronic micropump. The injector cannula was left in the guide cannula for an additional 5 minutes after injection to prevent flow into the cannula track, after which the injector cannula was removed and the dummy was put back in the guide cannula. Mice were returned to their home cage after each injection. Mice were injected three times for experiment 1: 1 hour before and 3 hours after the fear conditioning trial on day 1, and 1 hour before the context retrieval trial on day 2. Mice were injected a single time for experiments 2 and 3: 1 hour (experiment 2) or 2 hours (experiment 3) before the fear conditioning trial on day 1.

### Fear conditioning

Fear conditioning was done during the light phase between 9 am – 5 pm. Each fear conditioning experiment consisted of 3 trials: one fear conditioning trial on day 1, one context fear retrieval trial on day 2, followed 3 hours later by a tone fear retrieval trial. For the fear conditioning trial mice were placed in a rectangular box with steel walls and a steel grid floor (Coulbourn Instruments; H10-11R-TC, 12″Wx10″Dx12″H). After a 3 minute baseline recording, a 20 second tone was presented (2800 Hz, 82 dB) that simultaneously ended with a 2 second footshock (0.7 mA). One minute after the end of the first tone-shock presentation a second similar tone-shock was presented, and 1 minute after the second tone-shock presentation mice were returned to their home cage. During the context fear retrieval trial on day 2, mice were placed for a 3 minute period in the same box used for the fear conditioning trial. For the tone fear retrieval trial on day 2, mice were placed in a square plastic box (Tupperware; 9″Wx9″Dx8″H) with black and white striped walls, bedding on the floor, and acetic acid scent. After a 3 minute baseline recording, a 20 second tone was presented similar to the tone used during the fear conditioning trial.

### Histology

After behavioral testing was completed all mice were anesthetized and injected with ink using the same method that was used for Abeta and control injections. The brains were dissected, and the presence of ink inside the lateral ventricle was verified. Mice that did not have ink in the lateral ventricle were excluded from further analysis. This resulted in the exclusion of 9 out of 56 mice (experiment 1: 5 out of 16; experiment 2: 2 out of 20; experiment 3: 2 out of 20).

### Analysis of freezing behavior

Freezing behavior was quantified using automated analysis of videos. A digital camera was mounted above each box used for fear conditioning, and connected to a computer with Actimetrics FreezeFrame software (Coulbourn Instruments). Videos were collected during behavioral testing and analyzed after completion of behavioral testing. Freezing was quantified with the FreezeFrame software (bout length of 1 sec) for a number of time intervals. Time intervals analyzed for the fear conditioning trial on day 1: 0–60 sec (minute 1; m1), 60–120 sec (minute 2; m2), 120–180 sec (minute 3; m3), 180–200 sec (tone 1; t1), 200–260 sec (inter-tone interval; iti), 260–280 sec (tone 2; t2), 280–340 sec (end). Time interval analyzed for the context fear retrieval trial on day 2: 60–180 sec (context). Time intervals analyzed for the tone retrieval trial on day 2: 60–180 sec (pre-tone), 180–200 sec (tone). The first minute of both retrieval trials was excluded from analysis, since mice show a short period of hyperactivity after being moved from their home cage to a new box. This period of hyperactivity interferes with freezing behavior and thereby prevents reliable expression of conditioned fear during the first minute. Statistical analysis was done using Student's unpaired t-tests to compare the control group with the Abeta group.

## Supporting Information

Figure S1 **Immunodepletion removes Abeta oligomers but not secreted amyloid-precursor protein.** Two images are shown that cover the entire lanes that were labeled with the 6E10 antibody. The two images were taken using different settings because of the large differences in the intensity of the bands in the upper versus lower part of the lanes. The immunoprecipitation resulted in the removal of Abeta oligomers but not of secreted amyloid-precursor protein (sAPP). The non-specific bands are most likely caused by the 4G8 antibody that was used for the immunoprecipitation.
(TIF)

Figure S2 **Comparison between the three control groups from experiment 1–3 and a group of mice that was not subjected to surgery and injection.** A) Repeated injections of the control solution in experiment 1 increased freezing during the context retrieval trial on day 2 compared with mice that were never injected. Since mice were restrained for a short time period every time the injector was placed in the guide cannula before injection, we propose that repeated restraint stress caused by the repeated injections might have caused a non-specific increase in freezing on day 2. B–C) A single injection of control solution before fear conditioning, as done in experiment 2 (B) and 3 (C), did not significantly change freezing scores during the two retrieval trials on day 2 as compared to mice not subjected to surgery and injection. Error bars are standard errors of means. * *P*<0.05.
(TIF)
