# Supplementary figures and images for: Natural Amyloid-Beta Oligomers Acutely Impair the Formation of a Contextual Fear Memory in Mice
Source: PLoS One. 2012 Jan 4;7(1):e29940. doi: 10.1371/journal.pone.0029940 (PMC3251597; doi:10.1371/journal.pone.0029940)

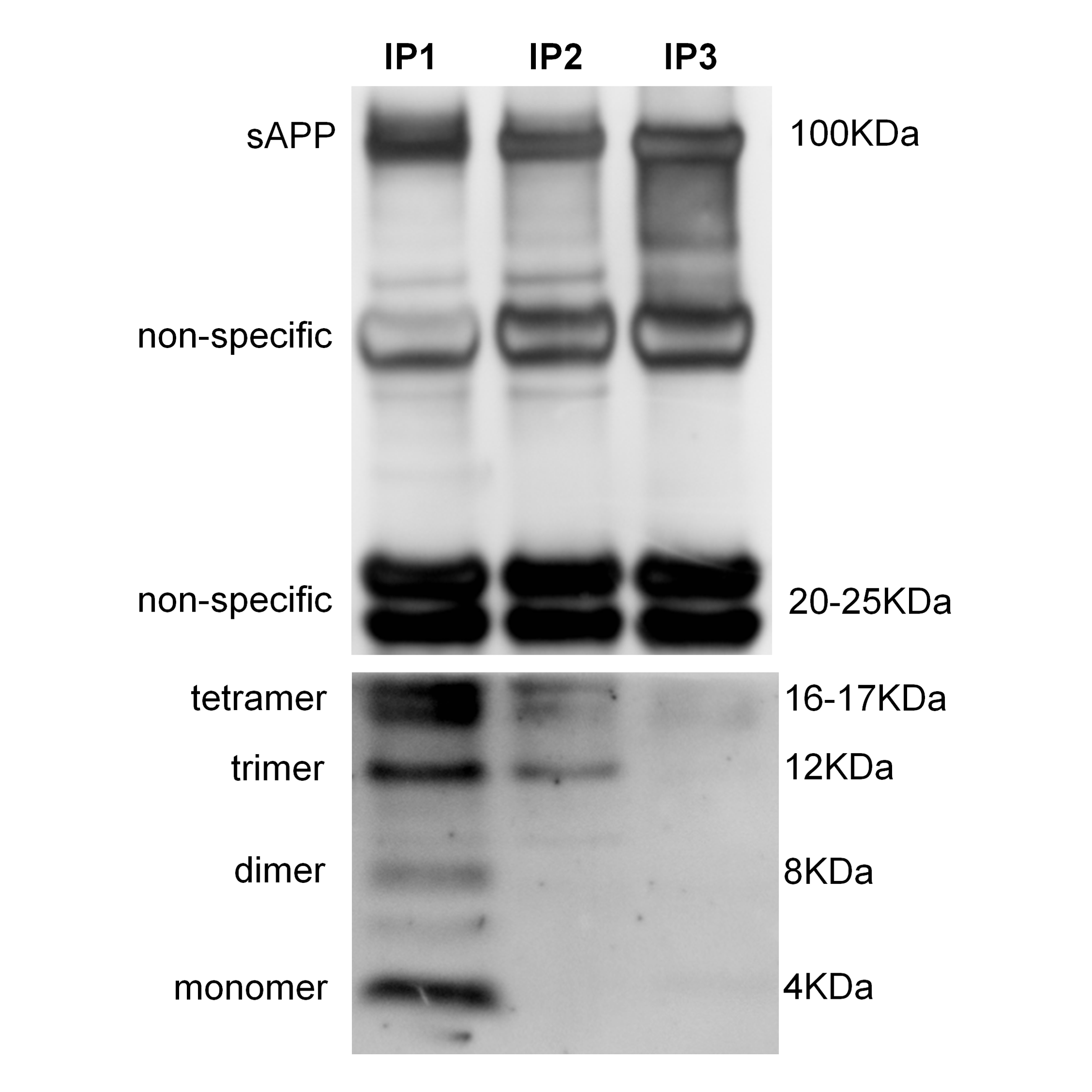

Supplement: Figure S1 — Immunodepletion removes Abeta oligomers but not secreted amyloid-precursor protein. Two images are shown that cover the entire lanes that were labeled with the 6E10 antibody. The two images were taken using different settings because of the large differences in the intensity of the bands in the upper versus lower part of the lanes. The immunoprecipitation resulted in the removal of Abeta oligomers but not of secreted amyloid-precursor protein (sAPP). The non-specific bands are most likely caused by the 4G8 antibody that was used for the immunoprecipitation. (TIF) [file pone.0029940.s001.tif]

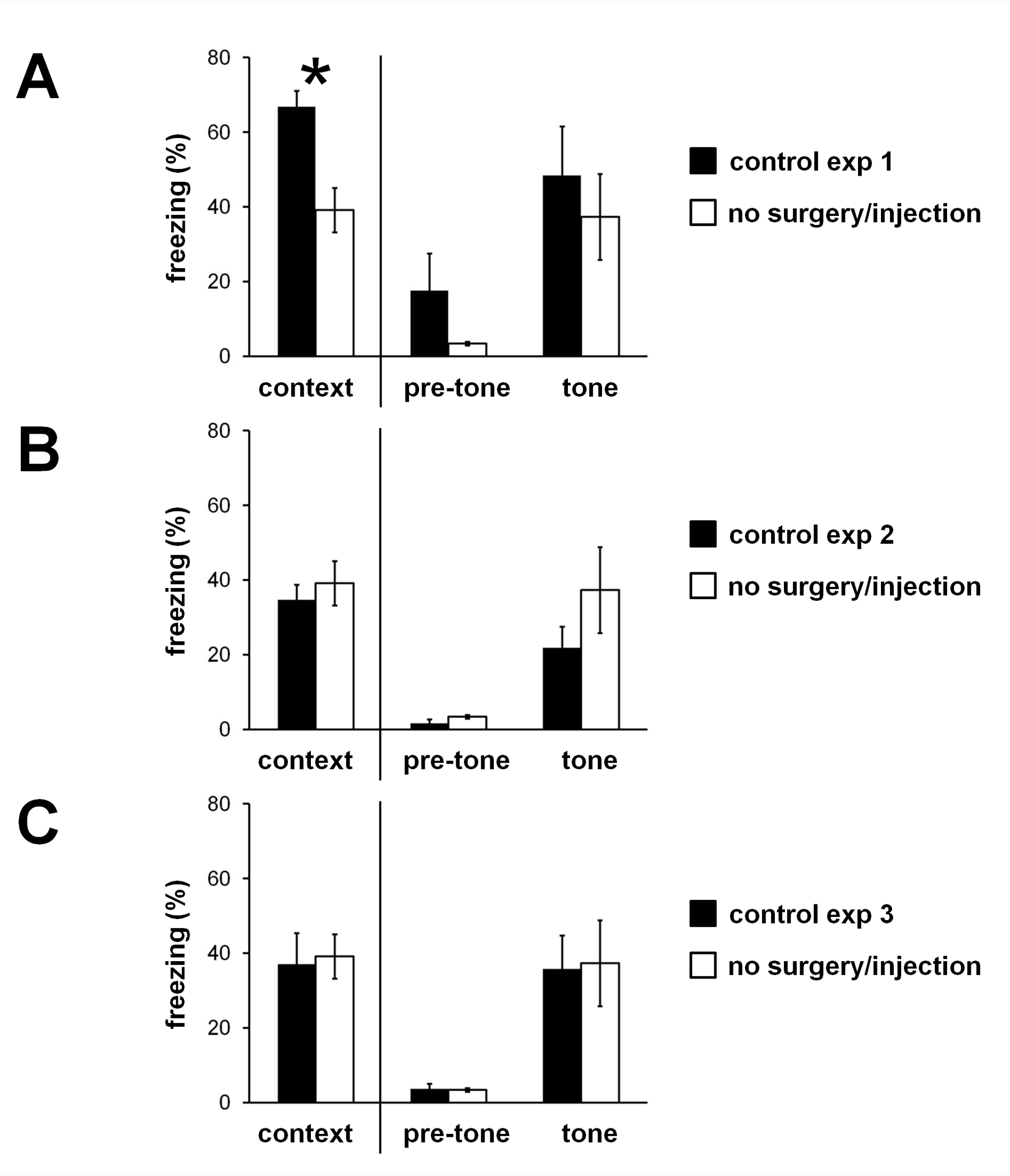

Supplement: Figure S2 — Comparison between the three control groups from experiment 1–3 and a group of mice that was not subjected to surgery and injection. A) Repeated injections of the control solution in experiment 1 increased freezing during the context retrieval trial on day 2 compared with mice that were never injected. Since mice were restrained for a short time period every time the injector was placed in the guide cannula before injection, we propose that repeated restraint stress caused by the repeated injections might have caused a non-specific increase in freezing on day 2. B–C) A single injection of control solution before fear conditioning, as done in experiment 2 (B) and 3 (C), did not significantly change freezing scores during the two retrieval trials on day 2 as compared to mice not subjected to surgery and injection. Error bars are standard errors of means. * P<0.05. (TIF) [file pone.0029940.s002.tif]
